# Relationships Between Annual and Perennial Seagrass (*Ruppia sinensis*) Populations and Their Sediment Geochemical Characteristics in the Yellow River Delta

**DOI:** 10.3389/fpls.2021.634199

**Published:** 2021-04-20

**Authors:** Ruiting Gu, Xiaoyue Song, Yi Zhou, Shaochun Xu, Shuai Xu, Shidong Yue, Yu Zhang, Xiaomei Zhang

**Affiliations:** ^1^CAS Key Laboratory of Marine Ecology and Environmental Sciences, Institute of Oceanology, Chinese Academy of Sciences, Qingdao, China; ^2^Laboratory for Marine Ecology and Environmental Science, Qingdao National Laboratory for Marine Science and Technology, Qingdao, China; ^3^College of Earth Sciences, University of Chinese Academy of Sciences, Beijing, China; ^4^Center for Ocean Mega-Science, Chinese Academy of Sciences, Qingdao, China; ^5^Key Laboratory of Marine Ecosystem Dynamics, Second Institute of Oceanography, Ministry of Natural Resources, Hangzhou, China; ^6^CAS Engineering Laboratory for Marine Ranching, Institute of Oceanology, Chinese Academy of Sciences, Qingdao, China

**Keywords:** submerged aquatic vegetation, *Ruppia*, population traits, sediment geochemical characteristics, sediment carbon cycling, sediment nutrients

## Abstract

Annual and perennial populations commonly occur for the same submerged aquatic angiosperm species, yet relationships between population types and sediment characteristics are poorly understood. In the current study two *Ruppia sinensis* habitats with annual and perennial populations were surveyed in the Yellow River Delta (YRD). Biomass and seasonal seed bank size were used to evaluate population status and potential recruitment capacity. Sediment geochemical parameters including moisture, sulfide, Chl *a*, carbohydrate, OM, TOC, TN, and TP were measured to compare sediment nutrient composition and variability. The results revealed a higher biomass and larger seed bank in the annual *R. sinensis* population compared with the perennial population. The P levels in sediments between the two *R. sinensis* populations were similar; while the N level in the sediment of the annual population was significantly higher than the perennial population, which might support the recruitment of vegetative shoots when a large amount of seeds germinated during wet periods. The annual population exhibited greater resilience after habitat desiccation, with the population recovering rapidly once water appeared. The results of this study add to the knowledge of *R. sinensis* populations and their sediment geochemical characteristics, and can be used as a reference for *Ruppia* population conservation and management.

## Introduction

The Yellow River Delta (YRD) is the broadest, and best conserved wetland ecosystem in temperate China (Han et al., 2006). Rich salt-tolerant plants are found in this area, with *Phragmites australis*, *Suaeda salsa*, and *Tamarix chinensis* being the dominant species (Zhang et al., 2013; Yu et al., 2016). Increasing anthropogenic activities, have resulted in the coastal wetlands of the YRD changing from natural wetlands to farmlands, and salt-culture ponds (Yu et al., 2012). These changes result in diverse sediment characteristics. For instance, the topsoil of *Deyeuxia angustifolia* wetlands contains more labile fraction organic carbon than an upland forest and two farmlands in the Sanjiang Plain of northeast China (Zhang et al., 2006). Meanwhile, the redistribution of local plants, including aquatic plants, have also caused shifts in the nutrient composition and chemical processes in the sediment, due to the close relationship between plants and sediment composition. For instance, *Thalassia testudinum* leaves are capable of inducing CaCO_3_ precipitation and increasing habitat sediment carbon storage (Enríquez and Schubert, 2014).

*Ruppia*, a genus of submerged marine angiosperms, commonly inhabit shallow systems, such as coastal lagoons and saltmarshes (Verhoeven, 1979; Mannino and Sara, 2006; Strazisar et al., 2015). Similar to other seagrass species, *Ruppia* species act as nursery areas for a variety of fishes and birds (Congdon and Mccomb, 1979; Rodriguez-Perez and Green, 2006; Mannino et al., 2015), provide food, increase water clarity by enhancing sedimentation (Barbier et al., 2011), and are key sites for global carbon storage (Fourqurean et al., 2012; Jiang et al., 2018). The high environmental adaptability of *Ruppia* not only leads to it being widely distributed around the world (Aedo and Casado, 1988; den Hartog et al., 2016; Martinez-Garrido et al., 2017), but also to high phenotypic plasticity, which characterizes the taxonomic confusion of this genus (Aedo and Casado, 1988; Yu and den Hartog, 2014). Yu and den Hartog (2014) recently updated the distribution and taxonomy of *Ruppia* in China and named two new species, *R. brevipedunculata* and *R. sinensis*, based on genetics and morphological characteristics. *R. sinensis* is widely distributed in abandoned salt pans and salt-culture ponds of the YRD (Gu et al., 2019) and forms dense, monospecific beds like other *Ruppia* species (Verhoeven, 1979; Mannino and Sara, 2006). Even though *R. sinensis* has the potential to be used as a phytoremediation species, little is known about the population characteristics of this species (Gu et al., 2020), and it is often ignored when researchers investigate plant distributions and calculate carbon storage (Zhang et al., 2013; Yu et al., 2016).

Annual or perennial life cycle strategies are commonly found in seagrass populations (Moore and Short, 2006). Sexual and asexual reproduction commonly occur in all seagrass populations, including annual and perennial populations (Sato et al., 2016; Entrambasaguas et al., 2017; Xu et al., 2018). Moreover, the life cycle strategy for seagrass individuals is considered to be genetically fixed, and annual and perennial seagrasses can be mixed within populations. Environmental factors, such as underwater photon flux density and the presence of water, have been recognized as major factors controlling seagrass survival, and may result in a seagrass population being perennial or annual (Kim et al., 2014; Mannino and Graziano, 2014). In perennial populations, the vegetative shoots of seagrass appear throughout the year and succession primarily relies on clonal reproduction (Verhoeven, 1979; Malea et al., 2004; Xu et al., 2018). In contrast, annual seagrass populations are absent during unfavorable conditions (e.g., freezing or desiccation) and re-establishment is completely dependent on seed germination (Verhoeven, 1979; Strazisar et al., 2013a, b). Many studies have suggested that these two kinds of shoots, perennial and annual, could be found in both perennial and temporary *Ruppia* populations. *Ruppia* populations with vegetative shoots present throughout the year are considered to be perennial populations. Populations exhibiting an annual life history complete their life cycle within a few months, and survive as seeds before producing the next generation of plants (Verhoeven, 1979; Brock, 1982; Malea et al., 2004; Mannino and Graziano, 2014). Many studies have compared the morphological variations in different *Ruppia* population types. Longer roots and bigger leaves are found in *R. maritima* and *R. cirrhosa* perennial populations, while more flowers are found in annual populations (Malea et al., 2004; Mannino and Graziano, 2014).

Sediment nutrition is vital for seagrasses and population strategy may affect sediment nutrition status. Phosphorus, nitrogen, sulfur, and carbon can be measured in the sediment to reveal the relationships between vegetation and sediment nutrition. Nitrogen and phosphorus are considered to be the two most important elements related to vegetative growth (Liao et al., 2008; Ailstock et al., 2010; Qu et al., 2018), and their concentrations in the sediment are strongly linked to seagrass biomass development and rapid recruitment (Menendez, 2009; Strazisar et al., 2013a,b). The reproductive organs of vegetative plants are often phosphorus-rich (Kerkhoff et al., 2006), while there are higher nitrogen requirements in photosynthesizing leaf tissue (Feller et al., 2008). Sulfide is considered toxic to seagrass. For instance, a direct link between high sediment sulfide levels and mortality of *T. testudinum* has been noted (Carlson et al., 1994). Total organic carbon (TOC), organic matter (OM), and carbohydrates are the three parameters used to describe the carbon sink in the sediment. TOC represents the quantity of buried organic carbon, whereas the compositional features of OM identify the source of organic matter (Geraldi et al., 2019; Kaal et al., 2020a, b). Carbohydrates are an important source of organic matter in the aquatic environment (Burdige et al., 2000), which mainly come from the microbial degradation of organic matter such as photosynthetic organisms and provides an index to assess recent sediment conditions (Bianchelli et al., 2016; Artifon et al., 2019).

In the current study, we selected two *R. sinensis* populations considered to be annual and perennial in the YRD. We recorded the seasonal changes of these two populations, and investigated their sediment characters, including inorganic nutrient concentrations and physical parameters. This study aimed to (1) elucidate the main factors resulting in *R. sinensis* populations being differentiated as annual or perennial and (2) assess the effect of *R. sinensis* absence on habitat sediments. The results of this study could serve as simple records for *Ruppia* populations in YRD, further our understanding of annual and perennial *Ruppia* characteristics and provide more information to inform *Ruppia* management.

## Materials and Methods

### Study Site

**FIGURE 1:**
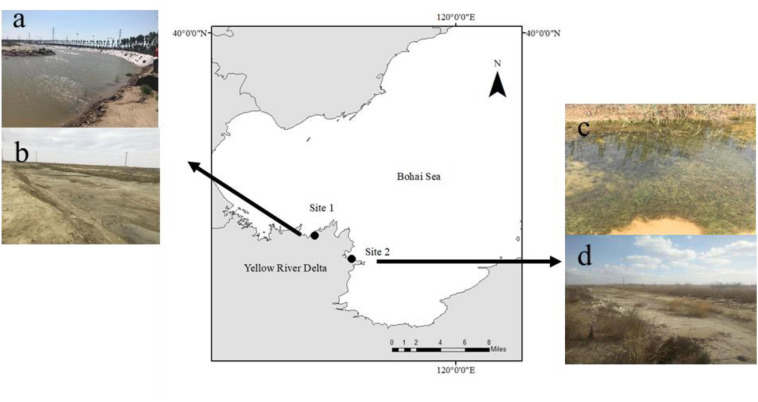
Study sites in the Yellow River Delta. **(a)** high water levels at Site 2 in August 2017; **(b)** extreme desiccation period at Site 2 in March 2018; **(c)** high water level at Site 1 in May 2017; and **(d)** dry season at Site 1 in December 2017.

The Yellow River Delta (YRD) is a wetland ecosystem in the warm temperate zone of China covering an area of approximately 5400 km^2^ (Yu et al., 2016). The YRD is considered a carbon sink hotspot due to the large amounts of particulate carbon that are transferred here (Zhao et al., 2020). Yu and den Hartog (2014) identified a new species, *R. sinensis*, which is widely distributed in the YRD (36°55′–38°16′, 117°31′–119°18′, Li et al., 2009). In this study, we selected two *R. sinensis* populations located 45 km apart (Figure 1), comprising an annual (Site 1) and perennial population (Site 2), respectively. Site 1 was a 1,200 m^2^ ditch, which was near the YRD Nature Reserve (37° 45′ 55.83″ N; 118° 58′ 13.03″ E). The water level at Site 1 fluctuates seasonally, becoming dry in winter. Site 1 has an annual *R. sinensis* population. Site 2 occurred around a brackish water pond (37° 59′ 52 N; 118° 36′ 33″ E) and was approximately 5,000 m^2^ in area. The salinity at the two sites ranged from 7.2–11.6 to 9.3–16.7 psu, respectively, and there was little anthropogenic influence at either site.

### Environmental Parameters and *R. sinensis* Populations

We investigated the two *R. sinensis* populations over a period of 2 years. This observation period included an unusual extreme desiccation event from March 2018 to May 2018 (Figures 1b,d), which resulted in both survey sites drying up. Environmental data including temperature and precipitation, were downloaded as daily records from Tianqi.2345.com^1^. Precipitation and evaporation are two of the main factors impacting the water levels of these two closed, natural *R. sinensis* habitats. Temperature is an indirect parameter that indicates water evaporation at the survey sites. The quantity of precipitation was used as an indirect parameter to indicate water input. Water temperature is closely related to air temperature in these shallow water systems. Thus, air temperature was used to represent the seasonal temperature in *R. sinensis* habitats, and weather records indirectly indicated the precipitation at the study sites. The mean monthly temperatures of the two sites were calculated to represent seasonal temperature changes. The weather records were divided into four categories: light rain or snow (precipitation < 10 mm/24 h, value = 1), showers or thunderstorms (value = 2), moderate rain or snow (10 mm/24 h < precipitation < 28 mm/24 h, value = 3), and heavy rain or rainstorm (precipitation > 25 mm/24 h, value = 4). We then quantified the monthly precipitation for each of these four precipitation categories.

The vegetative status of both *R. sinensis* populations were observed from October 2016 to July 2018 (observations were recorded in October and December 2016; March, May, June, August, October, and December 2017; and March, May, and July 2018). We randomly sampled four *R. sinensis* cores (diameter = 10.5 cm, depth = 10 cm, sample interval = 5 m) from May to August 2017 to compare the biomass of the two populations. The recruitment capacity of the two populations was assessed using seed bank size. To do this, we surveyed dynamic seed bank changes at the two sites using four replicate core samples (diameter = 6 cm, depth = 20 cm).

### Sediment Sample Collection

Sediment cores at two different depths (deep and shallow) were collected from the two sites. Four deep sediment cores (diameter = 4 cm, depth = 60 cm, sample interval = 2 m) were collected at each site in May 2017 and 2018, respectively, to investigate the vertical sediment characteristics in the two *R. sinensis* populations. For each deep sediment core, the upper 40 cm was analyzed, which was cut into 2 cm segments (a total of 20 subsamples) using a cutting ring and packaged into ziplock bags. The seasonal differences in the sediments at the two sites were investigated through four shallow sediment cores (diameter = 6 cm, depth = 35 cm, sample interval = 2 m) collected at five sampling periods, May, October, and December 2017, and May and July 2018. Each shallow core (upper 20 cm) was divided into 5 cm segments (a total of 4 subsample) and packaged into ziplock bags. All of the sediment samples were transported to the laboratory within 2 days (stored on ice to avoid the water in the samples evaporating during transportation), where they were stored at −20°C prior to sample analysis.

### Deep Sediment Core Analysis

Sulfide content, Chlorophyll *a* (Chl *a*), moisture content, carbohydrate, OM, TOC, total nitrogen (TN), and total phosphorus (TP) were measured in every second segment of the deep sediment cores (10 segments from each deep sediment core). Sediment grain size was determined by pooling five segments, i.e.,10 cm/sample, with three replicates. The sulfide content was determined using the iodometric method (National Marine Environmental Monitoring Center (NMEMC), 2007), and Chl *a* was analyzed fluorometrically following the Welschmeyer method (Welschmeyer, 1994). Pigments were extracted with 90% acetone over a period of 36 hours in the dark at 4 °C. The samples were centrifuged at 3,000 rpm for 15 min and the supernatant was used to determine the Chl *a* content. The rest of the sediment samples were freeze-dried. The moisture content (MC; %) of the sediment samples was determined using the following equation:

$$M\,C=\frac{M_{W}-M_{D}}{M_{W}}\times 100\%$$

where M_*W*_ represents the fresh weight (g) of the initial sediment, and M_*D*_ represents the weight (g) of the dried sediment. The dried sediment samples were sieved through a 250-μm sieve, after being ground by hand with a mortar, to remove coarse debris and stones. A number of analyses were then conducted to determine the physical and chemical properties of the sediments. Carbohydrates were analyzed using the phenol-sulfuric acid method (Gerchako and Hatcher, 1972) and expressed as glucose equivalents. OM was determined as the difference between the dry weight of the sediment and the residue left after combustion at 450°C for 4 h (Parker, 1983). Before TOC analysis, the sediment samples were treated with an excess of 10% HCl to remove carbonates that could interfere with TOC measurement (Hedges and Stern, 1984). TOC and TN content was then measured using a VARIO EL III elemental analyzer. TP was measured using the method modified for particulate TP determination (Zhou et al., 2003). The grain sizes were measured using the 10 cm sections of sediment examined through a Laser Particle Size Analyzer. The sediment type was determined by considering the proportion of clay (C) in the silt (S) as follows:

$$C/S=\frac{C\,r}{S\,r}$$

where, Cr represents the clay accumulation rate in the sediment sub-sample, and Sr represents the silt accumulation rate in the sediment sub-sample. The sediment is considered to be clay when C/S > 2, mud when 0.5 < C/S < 2, and silt when C/S < 0.5 (Folk et al., 1970).

### Shallow Sediment Core Analysis

The shallow sediment cores were each divided into four 5 cm segments. Each of these sub-samples was analyzed for moisture content, carbohydrate, OM, TOC, TN, and TP using the same analysis as described for the deep cores.

### Statistical Analysis

A three-way analysis of variance (three-way ANOVA) was employed to compare the general effects of population type (sample site), sediment vertical distribution (sample depth), and season (sample time) on the sediment indexes including moisture content, TP, TN, OM, carbohydrate, TOC, Chl *a*, and sulfide. When the interaction between sample time, sediment depth, and site was significant, a one-way ANOVA with Tukey’s multiple comparison was conducted to compare their effects (*p* < 0.05). The general differences between the two sites and the monthly differences of each site in terms of biomass (2017: May to August) and seed density (2017: March, May; October, December; 2018: May) were compared using a one-way ANOVA. The grain sizes including medium diameter (D_50_) and the clay/silt ratio at every sediment depth of the two populations were compared with one-way ANOVA. Principal component analysis (PCA) was used to assess the relationship between the sediment characteristics and the variables investigated including sample time, sediment depth, and sample site. PCA was performed using the “prcomp” function in the R software program to determine the multivariate ordination of the 11 sediment parameters for seasonal and vertical sediment assessment, and PCA plots were constructed using the FAC-TOEXTRA package (Kassambara and Mundt, 2017) in the R software program.

## Results

### *Ruppia sinensis* at the Two Study Sites

**FIGURE 2:**
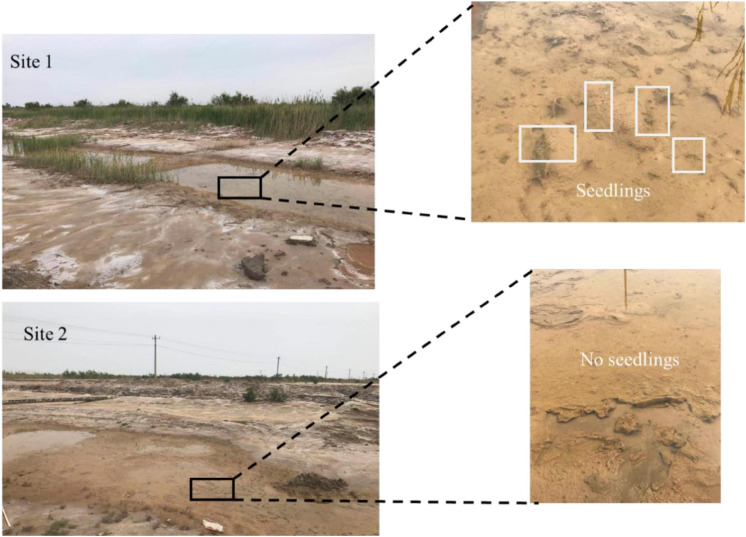
*Ruppia sinensis* growth status in May 2018.

The *R. sinensis* at both study sites differed in terms of population type (Table 1). The *R. sinensis* at Site 1 was an annual population, with vegetative shoots dying off each winter when the habitat became dry. Increasing precipitation and an accumulation of water in the habitat facilitated seed germination and the start of a new growing cycle. Although the surface water at Site 2 froze over in December 2017, the vegetative shoots survived, and green *R. sinensis* shoots could be observed under the ice layer. There was no significant difference in monthly population biomass between the two *R. sinensis* populations from May to August 2017 (*p*_*site*1_ = 0.174, *p*_*site 2*_ = 0.346). Although the monthly biomass at Site 1 was higher than at Site 2, these differences were not statistically significant, with the exception of May 2017 (*p*_*May 2017*_ = 0.03, Table 1). The seed densities at Site 1 were significantly higher than at Site 2 (*p* < 0.05, Table 1). An unexpected desiccation event occurred in December 2017, which caused the *R. sinensis* habitats at both sites to dry up until April 2018, with a small amount of water recorded in May 2018. Once water was present, the *R. sinensis* seeds at Site 1 germinated quickly, and several seedlings were observed. In contrast, no seedlings were observed at Site 2 when the water level increased (Figure 2). Both sites dried up again in July 2018 and all the vegetation died.

**TABLE 1:** Seasonal changes in *R. sinensis* populations and water levels at two study sites.

	Survey time	Water level		Biomass (g/m^2^)		Seed density (seeds/m^2^)	
		Site1	Site 2	Site1	Site 2	Site1	Site 2
2016	October	High	High	reproductive shoots	reproductive shoots	—	—
	December	None	Low	0	adult shoots	—	—
2017	March	High	High	—	—	109814 ± 15659*	4836 ± 1924
	May	High	High	367.06 ± 54.84*	206.21 ± 61.66	59684 ± 19669*	5662 ± 433
	June	High	High	439.51 ± 107.62	282.75 ± 138.40	—	—
	August	High	High	220.93 ± 65.49	151.25 ± 7.19	—	—
	October	Low	Low	short shoots	flowering and immature reproductive shoots	113470 ± 19810*	18519 ± 5819
	December	None	Low	0	adult shoots	112703 ± 22736*	26008 ± 9247
2018	March	None	None	0	0	—	—
	May	Low	Low	29.22 ± 9.56	0	39278 ± 3148*	26362 ± 3134
	July	None	None	0	0	—	—

*Indicates significant differences between the two sites, *p* < 0.05; “—” represents no investigative data for the corresponding month; Water level “High” indicates water level > 20 cm; “Low” represents ≤20 cm; “None” represents no flooding water.

### Relationship Between Sediment Nutrients and *R. sinensis* Growth

**FIGURE 3:**
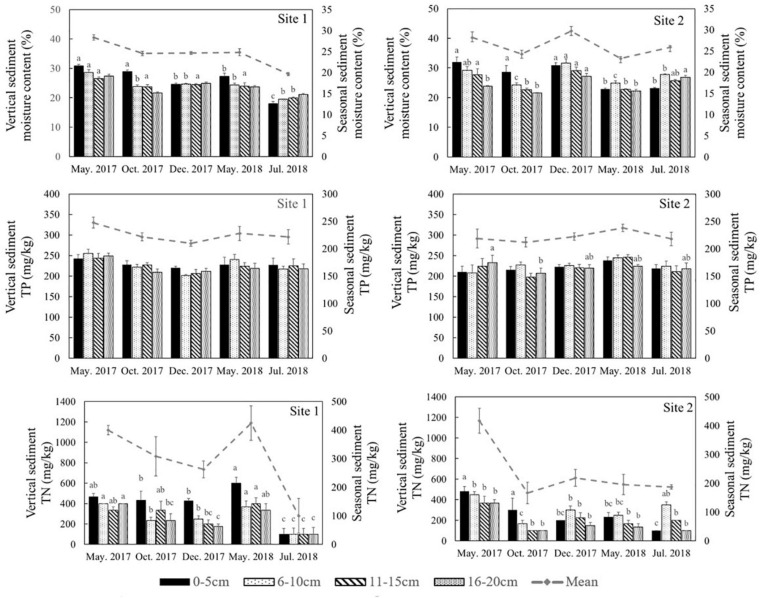
Moisture content, total phosphorus (TP) and total nitrogen (TN) in different sediment layers in different seasons at the two sites in the Yellow River Delta. The dotted lines represent the seasonal mean values of all parameters. Different letters represent significant differences between different seasons in the sediment (*p* < 0.05).

The environmental conditions of the two study sites, including temperature (evaporation) and rainy value (precipitation), were similar (Supplementary Figure 1). The highest mean temperature in this area during the study period was 28.5°C in July, while the lowest mean temperature was −2°C in January (Supplementary Figure 1A). The two sites had similar mean annual rainfall levels of 192 and 193 mm, respectively. The monthly precipitation at the two sites showed significant seasonal differences (Supplementary Figure 1B), with May to July being the three wettest months. In general, the sediment grain sizes at both sites were similar, indicating similar water storage capacities at the two sites (Table 2), though there was a slight difference in their vertical composition. The moisture content of the top 5 cm of the sediments was closely correlated with the appearance of water (Figure 3) and there was significant seasonal variation at both sites (Supplementary Table 1, *p* < 0.001). In 2017, the surface sediment contained more water than the deeper layers. In contrast, the deeper layers of sediment were wetter than the surface sediment in 2018, which was the dry year (Figure 3).

**TABLE 2:** Grain sizes at different sediment depths at the two study sites in the Yellow River Delta.

Depth	D_50_		Clay / Silt Ratio		Type of sediment	
	Site 1	Site 2	Site 1	Site 2	Site 1	Site 2
1-10 cm	24.07 ± 1.53	27.36 ± 4.57	3.61 ± 0.28	2.45 ± 0.51	Clay	Clay
11-20 cm	31.09 ± 5.00	33.58 ± 0.82	2.42 ± 0.88	1.80 ± 0.08	Clay	Mud
21-30 cm	26.43 ± 1.40	37.85 ± 2.36	3.41 ± 0.52	1.39 ± 0.23	Clay	Mud
31-40 cm	27.62 ± 1.86	25.20 ± 4.28	3.88 ± 0.32	1.59 ± 0.81	Clay	Mud

**FIGURE 4:**
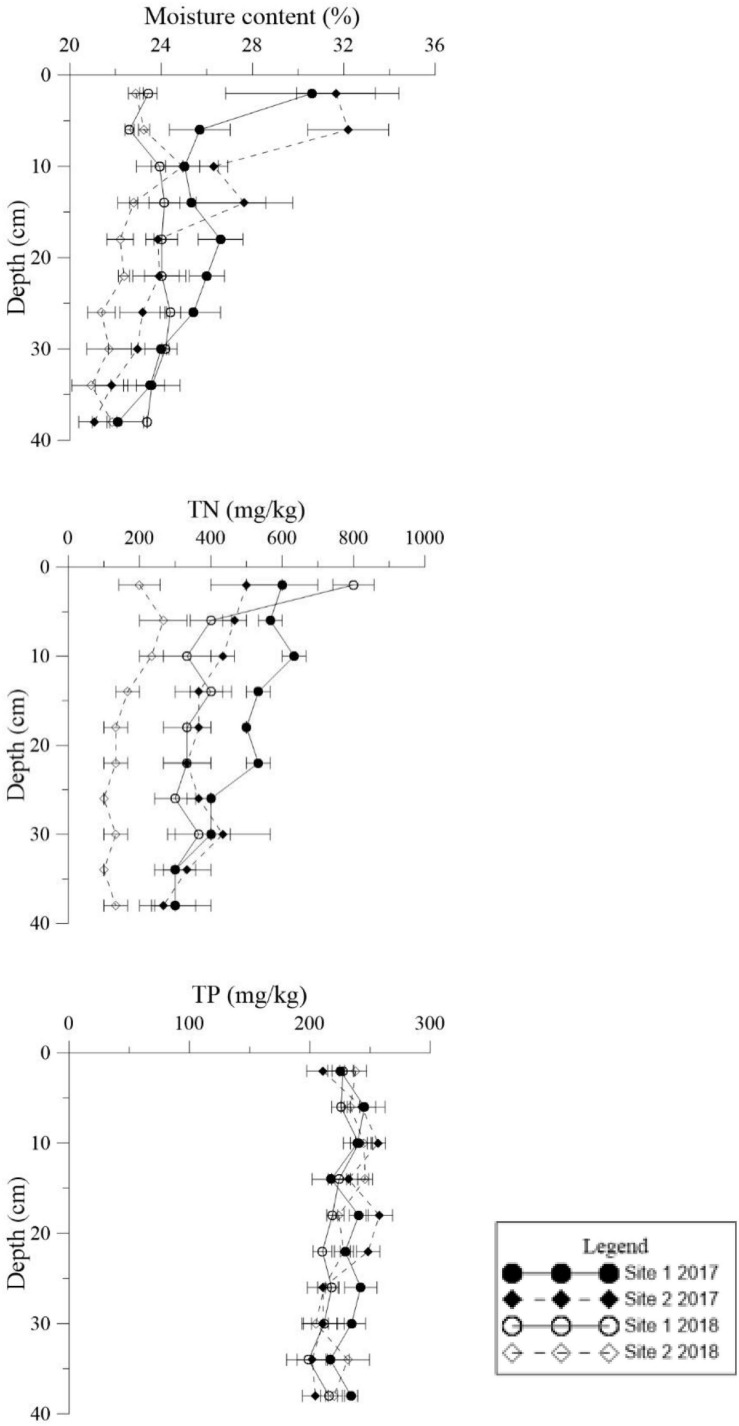
Vertical distributions of moisture content, total phosphorus (TP), and total nitrogen (TN) in the deep sediment cores from the two study sites in May 2017 and May 2018.

**FIGURE 5:**
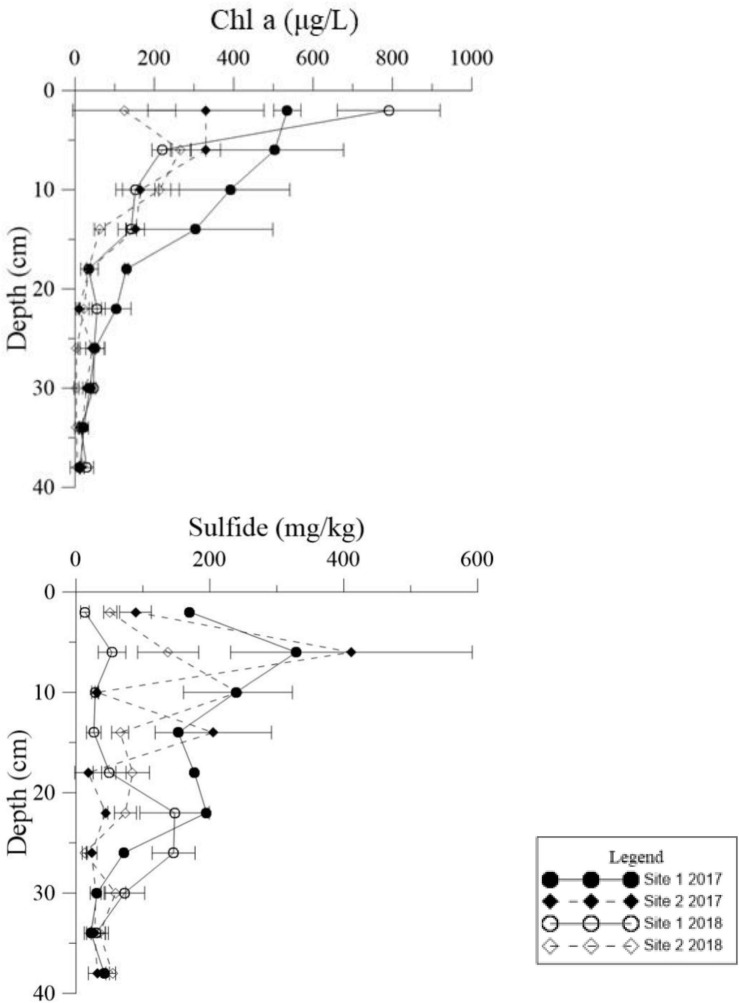
Vertical distributions of Chl *a* and sulfide in the deep sediment cores from the two study sites in May 2017 and May 2018.

Similar levels of TP were found at both sites (*p* = 0.466, Supplementary Table 1), although they showed slight seasonal variations. In general, TN in the sediment at Site 1 was higher than the sediment at Site 2 (Figure 3, *p* < 0.001). In 2018 TN was lower than 2017 levels at both sites (Figure 4, *p* < 0.001). TN in the surface sediment of Site 1 was higher than that of the other sediment layers; however, this pattern was not observed at Site 2 (Figure 3). Higher levels of TN were recorded during the vigorous growth period of *R. sinensis*, which occurred at both sites in May 2017 and at Site 1 in May 2018 (Figure 3). This changing trend was closely correlated with the appearance of *R. sinensis* (Table 1). Highest levels of sulfides were found at a depth of 6–10 cm at both sites, and sulfide levels in sediments deeper than 14 cm were not statistically different to the top 2 cm (Figure 5, *p* < 0.001), indicating that 6–14 cm was the most suitable sediment layer for sulfide accumulation.

### The Relationship Between *R. sinensis* Shoot Decomposition and Sediment Composition

**FIGURE 6:**
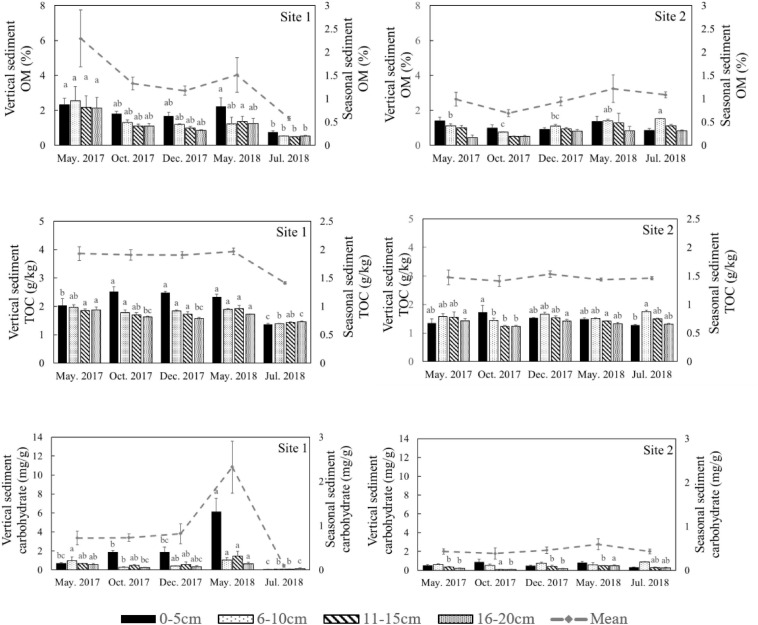
Organic matter (OM), total organic carbon (TOC), and carbohydrate content in different sediment layers in different seasons at the two study sites. The dotted lines represent the seasonal mean values of all parameters. Different letters represent significant differences between different seasons (*p* < 0.05).

**FIGURE 7:**
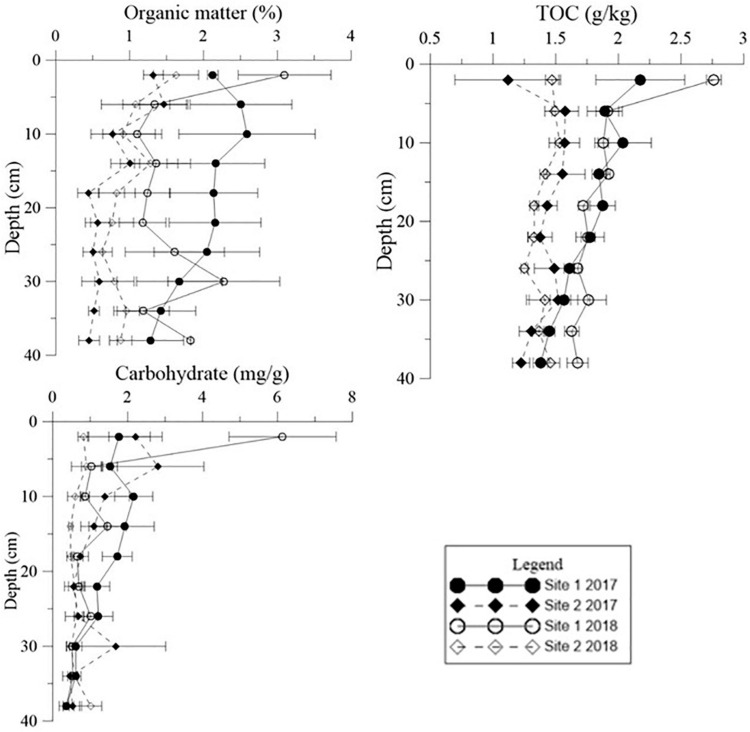
Vertical distribution of organic matter (OM), carbohydrate levels, and total organic carbon (TOC) in the deep sediment cores from the two study sites in May 2017 and May 2018.

Both Chl *a* and sulfide were measured in the deep sediment cores from both sites. Sediment layers deeper than 18 cm had low Chl *a* values at both sites (Figure 5, *p* < 0.001). During the regular year (2017), the Chl *a* level in the sediment at Site 1 was higher than at Site 2, which was correlated with the biomass differences between the two sites (Table 1). The Chl *a* levels in the sediment at Site 2 in 2017 were significantly higher than 2018; however there was no significant annual variation at Site 1. This may have been due to the disappearance of *R. sinensis* at Site 2 in 2018, while the *R. sinensis* population at Site 1 recovered rapidly. (Figure 5, *p*_*site 1*_ = 0.139, *p*_*site 2*_ = 0.012). There were seasonal changes in the three sediment carbon parameters (carbohydrates, TOC, and OM), and these changes differed between the two sites (Figure 6). While seasonal changes in OM content were observed at both sites, Site 1 had higher OM values than Site 2, corresponding with the presence of the *R. sinensis* population in May 2017 and 2018. No particular sediment layer exhibited a distinctive OM content, which indicated that its vertical distribution in these two sites was relatively balanced (Figure 7). The TOC at Site 1 was generally higher than Site 2. In contrast to OM, seasonal changes in TOC at both sites showed no significant seasonal differences, except for the sharp decrease in TOC when the water dried up in July 2018 at Site 1 (Figure 6). Moreover, the significant annual differences in TOC were only observed in the surface sediment, above 6 cm (Figure 7). In most seasons, there were higher levels of carbohydrates in the surface sediment (0-5 cm) of Site 1 than the deeper sediment layers (5-20 cm) (Figure 6, *p* < 0.001). Carbohydrate levels at Site 1 showed significant seasonal differences, which were twice as high as Site 2 (Supplementary Table 1, *p* < 0.001). The highest carbohydrate level at Site 1 was observed in May 2018, when water was present again and new *R. sinensis* seedlings were growing. No significant changes in carbohydrate values were found at Site 2 at this point.

### Sediment Assessment Index

**FIGURE 8:**
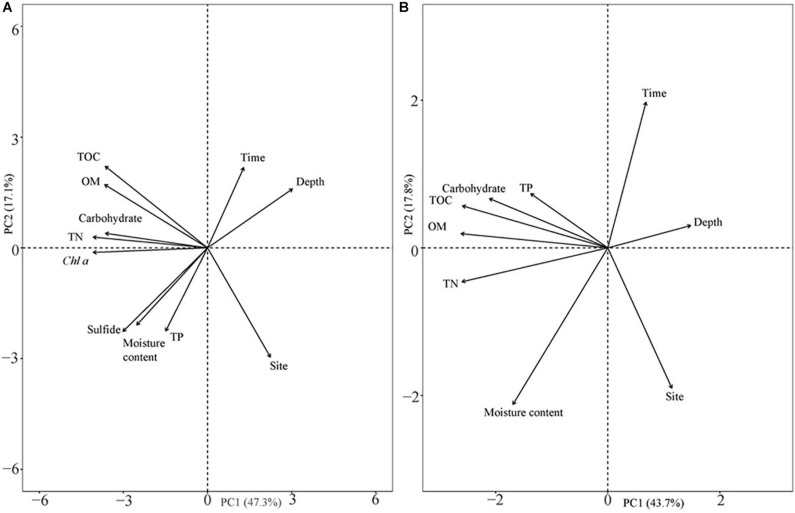
Principal component analysis of sediment parameters in different seasons and their vertical distribution. **(A)** Eight sediment parameters in deep sediment cores with annual differences and different vertical distributions in two different years; and **(B)** six sediment parameters in shallow sediment cores with seasonal changes and vertical distribution at the two sites.

The analysis results of the deep sediment cores with eight sediment assessment parameters (TOC, OM, carbohydrate, TN, Chl a, sulfide, moisture content, and TP) and three classifying parameters (time, depth, and site) explained 64.4% of the variation in the first two components (Figure 8A). The first component (PC1) represented 47.3% of the variance, while the second component (PC2) represented 17.1% of the variability and was dominated by different sample sites. Sulfide levels were closely related to the moisture content of the sediment (Figure 8A). Sampling site, which represented the different population types, was the highest contributing factor for variation in sediment characteristics in the deep cores with an annual sampling interval (Supplementary Table 2). In addition, in both shallow and deep cores, TOC contributed more than OM and carbohydrates when explaining the variation (Supplementary Table 3).

## Discussion

*Ruppia sinensis* is a commonly distributed seagrass in the YRD. Information relating to population types and the relationships between sediment characteristics and vegetative status are essential for population management. Similar to *R. cirrhosa* and *R. maritima* (Malea et al., 2004; Mannino and Graziano, 2014), annual and perennial traits occurred in the two *R. sinensis* populations in the YRD in the current study. Temperature and light availability are two key environmental parameters impacting the phenology of seagrass (Qin et al., 2020; von Staats et al., 2021). The presence of water in the habitat is another important factor that impacts the life cycle of this aquatic plant (Malea et al., 2004). In our previous study, around 70% of *R. sinensis* seeds collected from Site 2 germinated immediately under optimal germination conditions, and the dormancy of the remaining seeds could be broken by low temperatures (Gu et al., 2018). However, after an unusual desiccation event in 2018, *R. sinensis* seeds at Site 1 germinated immediately when surface water appeared, while no seedlings appeared at Site 2 (Figure 2). It appears that annual populations have higher stress resilience and a quick re-establishment capacity compared with perennial populations.

Although perennial populations of other *Ruppia* species are more reliant on clonal growth for population regeneration, both annual and perennial populations flower and produce seeds in the reproduction season (Mannino and Graziano, 2014). The larger seed densities of the *R. sinensis* annual population in the current study suggested that this population placed more reproductive energy into producing seeds than the perennial population (Table 1). However, similar to *Ruppia* habitats in the Everglades-Florida Bay ecotone (Strazisar et al., 2016), TP in the YRD was limited, and was not the main factor resulting in the two different *R. sinensis* life cycles. The TN levels in the sediment at Site 1 were higher than Site 2, which likely benefited the growth of shallow shoots, increasing the capacity for clonal growth within the population (Feller et al., 2008). In addition, the higher biomass of *R. sinensis* at Site 1 might also result in more TN input after decomposition of plant material at the end of the life cycle. The higher nutrient levels in the sediment of the annual *R. sinensis* population might support the recruitment of vegetative shoots when a large amount of seeds germinated during wet periods. In contrast, the nutrient conditions in the sediment of the perennial population were relatively stable.

Detritus from aquatic macrophytes is one of the most important endogenous sources of nutrients in wetlands (Wu, 2009). The Chl *a* content of sediments is a good representation of the abundance of primary producers such as living algae and undegraded macrophyte tissues (Mannino and Sara, 2006; Pusceddu et al., 2009). Low Chl *a* levels below depths of 18 cm in the sediments could imply limited presence of algal and *R. sinensis* detritus at this depth. However, in the shallow sediment layers, higher Chl *a* values were found when more *R. sinensis* was growing, which may be a result of more *R. sinensis* detritus settling in sediment and more suitable conditions for algae growth at this time. Moreover, a previous study indicated that *Ruppia* not only provided organic carbon directly to the biogeochemical cycle but also provided physical support for the attachment of other macrophytes, which also amplified the production and diversity of the system (Mannino and Sara, 2006). Sediment sulfide content was also closely related to organic deposition. A sharp decrease in *R. sinensis* biomass occurred at both sites after the dry spell, which may have resulted in a decrease in sediment sulfide levels. The results of the current study also showed a peak in the vertical sulfide gradient at a depth of 6 cm, indicating that the sediment conditions at this depth were suitable for sulfide accumulation (Figure 5).

The organic carbon from *Ruppia* detritus supports a complex food web through bacterial decomposition (Mannino and Sara, 2006). Organic carbon also indicates the quantity of leaf decomposition which increases nutrient supply for the survival of vegetation (Fan et al., 2015). The three different carbon parameters used in the current study showed slightly different trends. OM decreased with the deposition of *R. sinensis* shoots, and then increased significantly when surface water appeared in May 2018, when *R. sinensis* seedlings germinated at Site 1. Although there was a similar trend in OM at Site 2, there was no germination of *R. sinensis* seeds. This variation indicated the presence of algae, which emerged quickly when surface water appeared and is also an important resource of sediment OM (Figures 2, 7). TOC content has previously been used to represent OM (Sardans et al., 2017). However, even though the correlations of these two parameters were similar (Figures 6, 8), the seasonal changes in these two parameters were slightly different. A recent study noted that the main components of available OM to biota in aquatic ecosystems are carbohydrates, lipids, and proteins (Dias et al., 2017). Meanwhile, carbohydrates are more closely related to phytoplankton origin and vegetal detritus (Cotano and Villate, 2006). The carbohydrate levels in the sediments of the two survey sites differed more than OM and TOC (Figure 6). This variation might be related to the appearance of vegetation, as the highest carbohydrate level at Site 1 was recorded in May 2018, when both algae and *R. sinensis* were rapidly growing. However, the carbohydrate levels in the sediment at Site 2 were always low (Figure 6).

In summary, the two different *R. sinensis* population types in the YRD exhibited different resilience strategies under extreme desiccation conditions. An annual *R. sinensis* population was present at Site 1. Higher levels of TN were observed at this site which could benefit *R. sinensis* seedling growth, and promote population recovery when the water re-accumulated after drying up in winter. The appearance of *R. sinensis* was also accompanied by more algal growth. This not only increased primary productivity, but also increased carbon deposition and enriched the sediment. The appearance of water was the key factor resulting in the two different *R. sinensis* population types, which could be represented through sediment characteristics such as water moisture content. Of the different carbon parameters used to evaluate sediment carbon deposition in the current study, TOC was the most indicative in explaining the differences between the different *R. sinensis* populations. There were higher TOC levels recorded in the annual *R. sinensis* population compared with the perennial population. The results of this study provided a useful reference for the conservation and management of both annual and perennial *Ruppia* populations. This study also provided an example of the sensitivity of different carbon parameters in assessing the relationships between vegetation and sediment carbon.

## Data Availability Statement

The raw data supporting the conclusions of this article will be made available by the authors, without undue reservation.

## Author Contributions

RG did the conceptualization, investigated the data, and composited the original manuscript. XS performed the methodology. YZ carried out the funding acquisition, supervised the data, and reviewed and edited the manuscript. ScX, SX, SY, YuZ, and XZ investigated the data. All authors contributed to the article and approved the submitted version.

## Conflict of Interest

The authors declare that the research was conducted in the absence of any commercial or financial relationships that could be construed as a potential conflict of interest.

## Footnotes

1 http://tianqi.2345.com/wea_history/70617.htm

## Abbreviations

ANOVA: analysis of variance
OM: organic matter
PCA: principal component analysis
TN: total nitrogen
TOC: total organic carbon
TP: total phosphorus
YRD: Yellow River Delta.
