# Supplementary material for: Relationships Between Annual and Perennial Seagrass (Ruppia sinensis) Populations and Their Sediment Geochemical Characteristics in the Yellow River Delta
Source: Front Plant Sci. 2021 Apr 20;12:634199. doi: 10.3389/fpls.2021.634199 (PMC8095395; doi:10.3389/fpls.2021.634199)
Supplement: Supplementary file 1 [file Presentation_1.pdf]

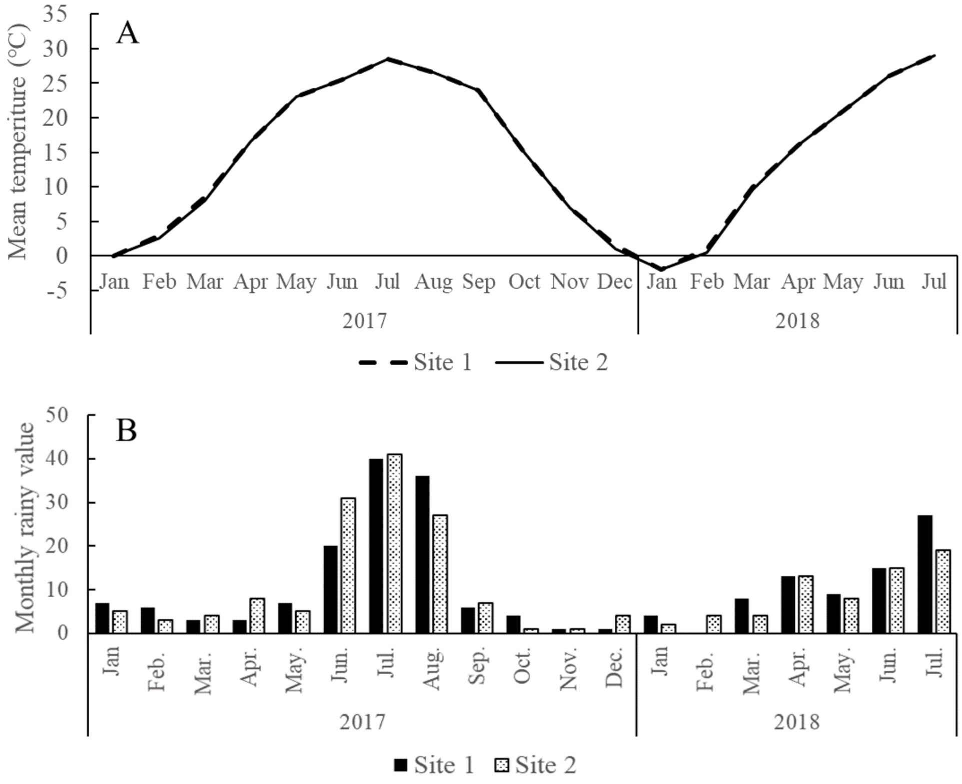

Supplementary Figure 1. Mean temperature (A) and mean precipitation (B) for the two study sites in the Yellow River Delta from 2017-2018.
