# Supplementary material for: Relationships Between Annual and Perennial Seagrass (Ruppia sinensis) Populations and Their Sediment Geochemical Characteristics in the Yellow River Delta
Source: Front Plant Sci. 2021 Apr 20;12:634199. doi: 10.3389/fpls.2021.634199 (PMC8095395; doi:10.3389/fpls.2021.634199)
Supplement: Supplementary file 2 [file Table_1.pdf]

Supplementary Table 1. Results of three- way ANOVA of shallow sediment cores

Variable	df	Sum square	Mean square	F-value	*p* (> F)	Variable	df	Sum square	Mean square	F-value	*p* (> F)
**Three-way ANOVA**											
**Moisture content (%)**						**OM (%)**					
Model	39	1728.18	44.312	16.232	< 0.001	Model	39	44.295	1.136	3.924	< 0.001
Depth	3	145.148	48.383	17.723	< 0.001	Depth	3	5.541	1.847	6.381	< 0.001
Time	4	621.945	155.486	56.957	< 0.001	Time	4	13.232	3.308	11.427	< 0.001
Site	1	218.041	218.041	79.871	< 0.001	Site	1	6.149	6.149	21.242	< 0.001
Depth * Time	12	375.471	31.289	11.462	< 0.001	Depth * Time	12	1.811	0.151	0.521	0.898
Depth * Site	3	35.153	11.718	4.292	0.006	Depth * Site	3	1.454	0.485	1.674	0.176
Time * Site	4	311.794	77.948	28.554	< 0.001	Time * Site	4	13.893	3.473	11.998	< 0.001
Depth * Time * Site	12	20.628	1.719	0.63	0.813	Depth * Time * Site	12	2.216	0.185	0.638	0.806
**TP (mg/kg)**						**Carbohydrate (mg/g)**					
Model	39	25230.658	646.94	1.611	0.026	Model	39	61.026	1.565	12.038	< 0.001
Depth	3	1206.891	402.297	1.002	0.395	Depth	3	12.645	4.215	32.426	< 0.001
Time	4	11347.968	2836.992	7.064	< 0.001	Time	4	13.367	3.342	25.709	< 0.001
Site	1	215.16	215.16	0.526	0.466	Site	1	3.914	3.914	30.109	< 0.001
Depth * Time	12	4996.785	416.399	1.037	0.42	Depth * Time	12	10.999	0.917	7.052	< 0.001
Depth * Site	3	971.706	323.902	0.807	0.493	Depth * Site	3	6.353	2.118	16.293	< 0.001
Time * Site	4	3759.453	939.863	2.34	0.059	Time * Site	4	7.418	1.855	14.268	< 0.001
Depth * Time * Site	12	2732.695	227.725	0.567	0.865	Depth * Time * Site	12	6.329	0.527	4.058	< 0.001
**TN (mg/kg)**						**TOC (g/kg)**					

Model	39	2764937.5	70895.833	16.764	<0.001	Model	39	15.254	0.391	21.859	<0.001
Depth	3	353520.83	117840.28	27.864	<0.001	Depth	3	2.153	0.718	40.108	<0.001
Time	4	1235527.8	308881.94	73.036	<0.001	Time	4	1.786	0.446	24.949	<0.001
Site	1	154173.61	154173.61	36.455	<0.001	Site	1	5.216	5.216	291.526	<0.001
Depth * Time	12	238250	19854.167	4.695	<0.001	Depth * Time	12	2.356	0.196	10.972	<0.001
Depth * Site	3	157020.83	52340.278	12.376	<0.001	Depth * Site	3	1.389	0.463	25.877	<0.001
Time * Site	4	505305.56	126326.39	29.87	<0.001	Time * Site	4	1.797	0.449	25.109	<0.001
Depth * Time * Site	12	121138.89	10094.907	2.387	0.008	Depth * Time * Site	12	0.557	0.046	2.594	0.004
