# Supplementary material for: Relationships Between Annual and Perennial Seagrass (Ruppia sinensis) Populations and Their Sediment Geochemical Characteristics in the Yellow River Delta
Source: Front Plant Sci. 2021 Apr 20;12:634199. doi: 10.3389/fpls.2021.634199 (PMC8095395; doi:10.3389/fpls.2021.634199)
Supplement: Supplementary file 3 [file Table_2.pdf]

Supplementary Table 2. Results of three- way ANOVA of deep sediment cores

Variable	df	Sum square	Mean square	F-value	*p (> F)*	Variable	df	Sum square	Mean square	F-value	*p (> F)*
**Three-way ANOVA**											
**Carbohydrate (mg/g)**						**Chl-a (μg/L)**					
Model	39	153.611	3.939	4.15	< 0.001	Model	39	4690280.1	120263.59	10.6	< 0.001
Time	1	3.422	3.422	3.605	0.06	Time	1	149733.77	149733.77	13.198	< 0.001
Depth	9	58.017	6.446	6.791	< 0.001	Depth	9	3555222.8	395024.75	34.819	< 0.001
Site	1	5.48	5.48	5.773	0.018	Site	1	242990.16	242990.16	21.418	< 0.001
Time × Depth	9	20.524	2.28	2.402	0.015	Time × Depth	9	169075.08	18786.12	1.656	0.107
Time × Site	1	3.664	3.664	3.861	0.052	Time × Site	1	5.333	5.333	0	0.983
Depth × Site	9	28.507	3.167	3.337	0.001	Depth × Site	9	299513.04	33279.227	2.933	0.004
Time × Depth × Site	9	33.997	3.777	3.98	< 0.001	Time × Depth × Site	9	273739.89	30415.543	2.681	0.007
**OM (%)**						**Sulfide (mg/kg)**					
Model	39	66.83	1.714	2.503	< 0.001	Model	39	1318182	33799.54	4.585	< 0.001
Time	1	0.046	0.046	0.067	0.796	Time	1	84250.109	84250.109	11.43	0.001
Depth	9	13.638	1.515	2.213	0.026	Depth	9	513079.28	57008.808	7.734	< 0.001
Site	1	31.87	31.87	46.549	< 0.001	Site	1	9478.975	9478.975	1.286	0.259
Time × Depth	9	6.334	0.704	1.028	0.422	Time × Depth	9	339674.79	37741.643	5.12	< 0.001
Time × Site	1	5.046	5.046	7.371	0.008	Time × Site	1	53263.354	53263.354	7.226	0.008
Depth × Site	9	3.018	0.335	0.49	0.879	Depth × Site	9	127459.03	14162.115	1.921	0.055
Time × Depth × Site	9	6.876	0.764	1.116	0.357	Time × Depth × Site	9	190976.51	21219612	2.879	0.004
**TN (mg/kg)**						**Moisture content (%)**					
Model	39	3812333.3	97752.137	17.25	< 0.001	Model	39	1043.292	26.751	4.686	< 0.001
Time	1	100277.78	1002777.8	176.961	< 0.001	Time	1	213.933	213.933	37.475	< 0.001

Depth	9	1000111.1	111123.46	19.61	< 0.001	Depth	9	339.778	37.753	6.613	< 0.001
Site	1	1002777.8	1002777.8	176.961	< 0.001	Site	1	18.709	18.709	3.277	0.073
Time × Depth	9	140555.56	15617.284	2.756	0.006	Time × Depth	9	270.515	30.057	5.265	< 0.001
Time × Site	1	186777.78	186777.78	32.961	< 0.001	Time × Site	1	20.376	20.376	3.569	0.061
Depth × Site	9	225000	25000	4.412	< 0.001	Depth × Site	9	143.853	15.984	2.8	0.005
Time × Depth × Site	9	254333.33	28259.259	4.987	< 0.001	Time × Depth × Site	9	36.129	4.014	0.703	0.705
**TOC (g/kg)**						**TP (mg/kg)**					
Model	39	14.196	0.364	12.473	< 0.001	Model	39	36319.371	931.266	1.784	0.009
Time	1	0.098	0.098	3.375	0.069	Time	1	1645.21	1645.21	3.152	0.078
Depth	9	3.012	0.335	11.47	< 0.001	Depth	9	15646.167	1738.463	3.331	0.001
Site	1	6.506	6.506	222.936	< 0.001	Site	1	175.85	175.85	0.337	0.563
Time × Depth	9	1.277	0.142	4.861	< 0.001	Time × Depth	9	6691.928	743.548	1.425	0.185
Time × Site	1	0.142	0.142	4.86	0.029	Time × Site	1	1943.131	1943.131	3.723	0.056
Depth × Site	9	2.973	0.33	11.319	< 0.001	Depth × Site	9	6760.198	751.133	1.439	0.179
Time × Depth × Site	9	0.188	0.021	0.717	0.693	Time × Depth × Site	9	3456.887	384.099	0.736	0.675
