# Supplementary material for: Relationships Between Annual and Perennial Seagrass (Ruppia sinensis) Populations and Their Sediment Geochemical Characteristics in the Yellow River Delta
Source: Front Plant Sci. 2021 Apr 20;12:634199. doi: 10.3389/fpls.2021.634199 (PMC8095395; doi:10.3389/fpls.2021.634199)
Supplement: Supplementary file 4 [file Table_3.pdf]

Supplementary Table 3. Parameters contributes to the PCs of PCA

	Dim 1(%)	Dim 2(%)
**Long cores**		
Time	1.564	12.263
Site	4.707	22.639
Depth	8.575	6.604
TOC	12.455	12.641
TP	2.087	13.102
TN	15.562	0.220
TOM	12.585	7.588
Carbohydrate	12.413	0.404
Moisture content	8.519	13.238
Chl a	15.560	0.039
Sulfide	5.972	11.263
**Shallow cores**		
Time	0.388	28.554
Site	6.535	0.673
Depth	3.902	16.425
TOC	8.545	32.923
TP	5.641	3.964
TN	13.303	3.272
TOM	20.449	0.272
Carbohydrate	20.188	1.540
Moisture content	20.051	2.377
